# Whole-genome-scale identification of novel non-protein-coding RNAs controlling cell proliferation and survival through a functional forward genetics strategy

**DOI:** 10.1038/s41598-021-03983-5

**Published:** 2022-01-07

**Authors:** D. P. Tonge, D. Darling, F. Farzaneh, G. T. Williams

**Affiliations:** 1 Faculty of Natural Sciences, School of Life Sciences, Keele University, Keele, ST5 5BG UK; 2 Molecular Medicine Group, Faculty of Life Sciences & Medicine, School of Cancer & Pharmaceutical Sciences, Kings College London, London, UK

**Keywords:** Non-coding RNAs, Cancer, Cell biology, Molecular biology

## Abstract

Identification of cell fate-controlling lncRNAs is essential to our understanding of molecular cell biology. Here we present a human genome-scale forward-genetics approach for the identification of lncRNAs based on gene function. This approach can identify genes that play a causal role, and immediately distinguish them from those that are differentially expressed but do not affect cell function. Our genome-scale library plus next-generation-sequencing and bioinformatic approach, radically upscales the breadth and rate of functional ncRNA discovery. Human gDNA was digested to produce a lentiviral expression library containing inserts in both sense and anti-sense orientation. The library was used to transduce human Jurkat T-leukaemic cells. Cell populations were selected using continuous culture ± anti-FAS IgM, and sequencing used to identify sequences controlling cell proliferation. This strategy resulted in the identification of thousands of new sequences based solely on their function including many ncRNAs previously identified as being able to modulate cell survival or to act as key cancer regulators such as AC084816.1*, *AC097103.2*, *AC087473.1*, CASC15*, DLEU1*, ENTPD1-AS1*, HULC*, MIRLET7BHG*, *PCAT-1*, *SChLAP1*, and *TP53TG1*. Independent validation confirmed 4 out of 5 sequences that were identified by this strategy, conferred a striking resistance to anti-FAS IgM-induced apoptosis.

## Introduction

Non-protein-coding sequences in the genome play crucial functional roles in a range of different cellular processes^1–47^. Most of the human genome encodes ncRNAs, most of which, at > 200 nucleotides, are classified as lncRNAs. Although the analysis of this vast number of transcripts is still at an early stage, it is evident that many lncRNAs play crucial roles in molecular cell biology, ranging from provision of essential frameworks for RNA processing, through to epigenetic control of gene expression, regulating cell signalling pathways, and more^46,48^. In the healthy state, lncRNA expression is often tightly restricted to specific tissues at specific times^48,49^. In particular, Sarropoulos et al.^50^ have demonstrated that many lncRNAs are dynamically regulated during organogenesis, displaying striking specificity both in tissue and chronological time of expression, emphasising their importance in regulating cell fate^50^. It is not surprising therefore that deregulation of lncRNAs has been implicated in pathology^51,52^, including oncogenesis^50,53–56^. Although demonstrations of the functional importance of individual RNAs are impressive and growing, the vast majority—many thousands—of lncRNAs are still entirely uncharacterised; some of these currently uncharacterised lncRNAs are likely to play critical roles in important control processes that have yet to be revealed. This provides strong motivation for the development of high-throughput strategies for the identification of key lncRNAs by targeting their functional activity.

For protein-coding genes, forward genetics approaches, such as unbiased gene modulation/mutation followed by selection for function, have already been widely applied in the identification of critical cell regulators^57^. In our own studies, functional cDNA expression cloning pinpointed not only several significant regulatory proteins^58,59^, but also 3 lncRNAs that modulate cell fate: *GAS5*^60,61^, *RBM5-AS1/Je2*^58,59^, and *NEAT1*/Trophoblast Derived Non-Protein Coding RNA^60,62^. All 3 lncRNAs were identified through the functional activity of short partial sequences, rather than the full-length RNA. In addition, a 24-base oligomer corresponding to part of *GAS5* also shows strong functional activity^63^. These results are all consistent with the observation that many lncRNAs act through short sequence motifs, although such functional sequences will often be required to act in concert^48,64,65^.

Here we describe a genome-scale strategy that builds upon these observations and identifies critical sequences through their functional effects on cell proliferation and survival. Our approach identifies those genes that play a causal role, and immediately distinguishes this group from those that are differentially expressed but do not affect cell function—this represents a key advantage over expression level studies. Furthermore, we use sequences derived from whole genomic DNA, thus overcoming the severe limitations imposed by the highly tissue-restricted expression of lncRNAs^46,50^. Our application of a powerful sequencing and bioinformatic approach complements an unbiased whole genome-scale screen, resulting in the identification of numerous novel ncRNA regulators with demonstrable function, as well as confirmation of the function of several known lncRNAs. In addition to this new information on genes with clear potential in physiology and pathology, these observations provide proof of principle for the application of this novel strategy to the identification of lncRNAs that play rate-limiting roles in many different cellular activities.

## Materials and methods

### Library preparation and culture selection methods

All mammalian cells were cultured at 37 °C, 5% CO_2_ in IMDM (Iscove’s Modified Dulbecco’s Medium; ThermoFisher #12440053) supplemented with 10% (v/v) Foetal Bovine Serum (FBS; Biosera FB-1001/500). Host cells were the anti-Fas-sensitive Jurkat T-leukaemic cell clone JKM1^66^.

Whole human genomic DNA (Promega G3041) was digested with the restriction enzymes Dra1 and Aan1 in two separate reactions. The resulting digests were combined at equimolar concentration to produce our initial “Genomic Digest” (i.e. restriction fragments with blunt termini from the whole human genome). The Genomic Digest was cloned into the Sma1 site of lentiviral expression vector pCDH-CMV4 (a third-generation lentiviral vector with deletion/fusion of the 5′ LTR and truncated (SIN) 3′ LTR); producing the expression library “CL3c” with inserts in both sense and antisense orientation. pCDH-CMV4 was a gift from Kazuhiro Oka (Addgene plasmid # 72284; http://n2t.net/addgene:72284; RRID: Addgene_72284). The library was introduced into MegaX DH10B electrocompetent cells (ThermoFisher) and amplified. For bulk production of the library in the lentiviral backbone, 8000 cm^2^ of tissue culture plasticware was plated at a density of 1.1 × 10^4^ 293T17 cells/cm^2^ in 1.4 L of DMEM + 10% FCS. After 72 h the cells were PEI transfected with 0.1 µg plasmid DNA per cm^2^ using a ratio of 2:1 PEI:DNA. The components of the transfection mix for lentiviral vector library synthesis were as follows: pMDG (14.6 ng/cm^2^), MDLg/pRRE (24.4 ng/cm^2^), pRSV-Rev (12.0 ng/cm^2^), pCL3c Vector plasmid (48.9 ng/cm^2^) in a total of approximately 2500 mL DMEM + 10% FCS/PEI/DNA mix. After 24 h incubation at 37 °C, 5% CO_2_, the medium was replaced with 2.4 L of DMEM + 10% FCS. After a further 24 h the crude lentiviral vector supernatant was harvested, 0.45 µm filtered and centrifuged for 18 h at 10,000×*g*, 6 °C. After centrifugation the supernatant was discarded, the lentiviral vector pellets resuspended in a total of 2.6 mL of Optimem and the concentrates aliquoted in 50 µL samples and stored at − 80 °C. The lentiviral library was transduced into Jurkat JKM1 cells at a multiplicity of infection of 3.7, i.e. 3.7 × 10^7^ virus constructs were introduced into each population of 10^7^ cells.

**Figure 1:**
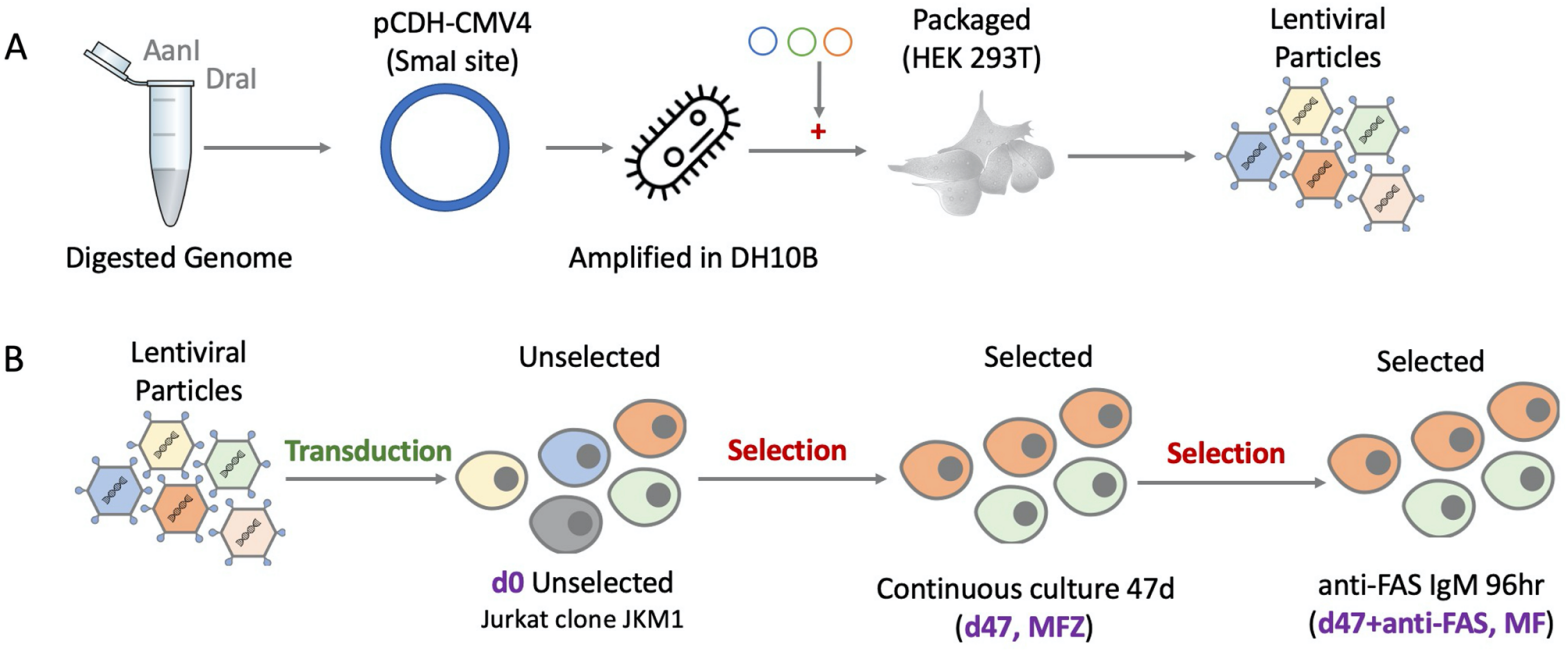
Experimental overview—(**A**) Human DNA was digested and cloned into pCDH-CMV4. (**B**) Jurkat clone JKM1 cells were transduced with our lentiviral library (CL3c) and harvested immediately following transduction (d0, Group = JCPZ), following 47 days of continuous culture (d47, Group = MFZ), or following 47 days of continuous culture followed by anti-FAS IgM selection for 96 h (d47 + anti-FAS, Group = MF). Four independent replicates were prepared for each condition (d0, d47 and d47 + anti-FAS).

Four replicate 50 mL Jurkat cultures (from 4 independent transductions) were maintained for up to 51 days, subculturing to 2 × 10^5^ cells/mL every 2–3 days. Samples of 5 × 10^6^ cells were taken immediately following transduction (to appraise the range of inserts successfully transduced relative to the starting library) (d0, JCPZ), at day 47 following continuous culture (d47, MFZ), and following 47 days of continuous culture plus additional selection with cytotoxic mouse IgM anti-Fas antibody IPO-4 at a final concentration of 20 ng/mL^58^ for 96 h at 37 °C (d47 + anti-FAS, MF). This d47 + anti-FAS, MF group was prepared by taking 4 × 10^7^ cells from each d47, MFZ replicate and subjecting each population to the additional selection with anti-FAS as described above. Surviving cells were harvested using Ficoll and their DNA isolated (Qiagen #69504). For schematic representation of these studies see Fig. 1.

### Sequencing and bioinformatic analysis

Extracted DNA was amplified by custom oligonucleotide primers targeting a region of the pCDH-CMV4 genome located adjacent to the Sma1 multiple cloning site, and thus designed to enable the amplification of all insert sequences irrespective of sequence composition (Forward: 5′ ccatccacgctgttttgacc 3′, Reverse: 5′ cgccggtacctttaagacca 3′). Amplicons were size selected using Ampure XP beads at a ratio of 1: 1.8 to remove unincorporated primers but retain the shortest products and sequenced using the Illumina platform with a 300 bp paired-end read metric.

**Figure 2:**
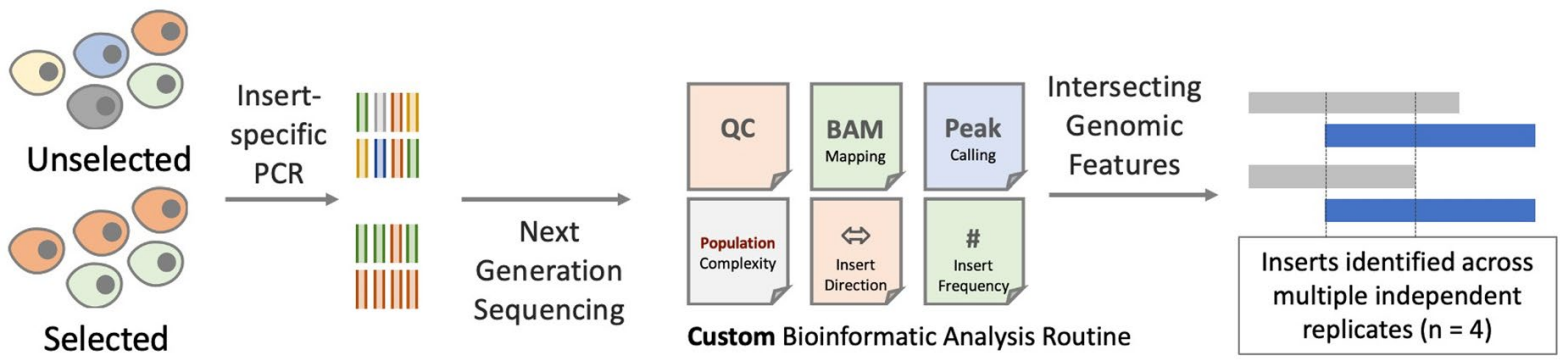
Bioinformatics overview—Insert sequences were amplified by PCR and sequenced using the Illumina HiSeq system with a 300 bp paired end read metric. A custom bioinformatic analysis workflow was developed to enable appraisal of the inserts within cells prior to (d0, JCPZ) and (in subsequent experiments) following selection (d47, labelled MFZ, and d47 + anti-FAS, labelled). This workflow validated inserts as “library-derived” through the presence of flanking vector sequence (recalling that the library was human in origin and transfected into human cells), confirmed the presence of an appropriate restriction site (DraI or AanI), and provided an indication of insert frequency, in addition to reporting the likely direction of insertion (and thus transcription). The presence of selected inserts across multiple experimental replicates was used to prioritise sequences for further validation.

A custom bioinformatic analysis workflow was developed to enable appraisal of the inserts within cells prior to (d0, JCPZ) and (in subsequent experiments) following selection (d47, labelled MFZ, and d47 + anti-FAS, labelled MF—see Fig. 1). This workflow validated inserts as “library-derived” through the presence of flanking vector sequence (recalling that the library was human in origin and transfected into human cells), confirmed the presence of an appropriate restriction site (DraI or AanI), and provided an indication of insert frequency, in addition to reporting the likely direction of insertion (and thus transcription). Sequencing reads were trimmed of low-quality bases and mapped to the latest human genome version (hg38) using BMA-MEM version 0.7.17.2 in simple Illumina mode. Areas of the genome enriched in mapped reads were identified by MACS2 version 2.1.1.20160309.6 and output as a list of genomic intervals in standard bed format^67^. Sequences located within 25 bp of each other were considered to originate from the same overall sequence and thus consecutive intervals within 25 bp of another were combined using bedtools merge (-d 25). The abundance of each insert was determined in each experimental sample using bedtools multicov where the number of reads mapped to each insert was used as a surrogate indicator of the number of cells harbouring that specific insert within a given cell population. We generated lists of overlapping inserts (based upon their genomic coordinates) across the different independently treated replicates using MultiIntersectBed^68^, and these data were used to identify candidate functional RNAs present in 3 or more independent replicates (Fig. 2). Our initial approach, reported herein, ensured that only very high-confidence hits (functional sequences identified in ≥ 3 independent replicates, plus evidence of vector associated sequence) were considered for further validation. Significant scope exists to appraise those hits present in less than 3 experimental replicates.

### Independent experimental validation of novel cell fate modifying sequences

Candidate sequences for independent validation (Table 7) were chemically synthesised and inserted into pcDNA3.1 sense expression plasmids (GenScript Limited). Constructs containing candidate sequences and control empty vector (pcDNA3.1 sense) were transfected into Jurkat JKM1 cells. The polyclonal populations were selected in 1 mg/mL geneticin for 14 days, grown to saturation density and challenged with 10–20 ng/mL anti-Fas IgM at 5 × 10^5^ cells/mL, in parallel with vector-only-transfected control cell cultures in the same 24-well plates. Viable (trypan-blue-excluding) cell densities were determined 7–18 days after addition of anti-Fas IgM.

## Results and discussion

### Development of the genome-scale library (CL3c) and mammalian cell transduction and functional selection

The observation that some lncRNAs can act through short sequence motifs led us to hypothesise that key functional potential could be revealed by expression of genomic DNA (i.e. in the absence of post-transcriptional processing). The first stage in this strategy was the preparation of a lentiviral library containing most of the human genome in both sense and antisense orientations and its transduction into the human T-leukaemic Jurkat clone JKM1, which is highly sensitive to apoptosis induced through ligation of the Fas receptor^66^ (see “Materials and methods” section). Commercially sourced human DNA was digested by the enzymes DraI and AanI to reduce the genome to a series of fragments that were predominantly in the predicted range of 0.5–4 kb. The resulting fragments were cloned into the pCDH-CMV4 vector in both orientations (the “CL3c library”), ensuring that transcript was generated from the entire genome from both the sense and anti-sense strands.

**Figure 3:**
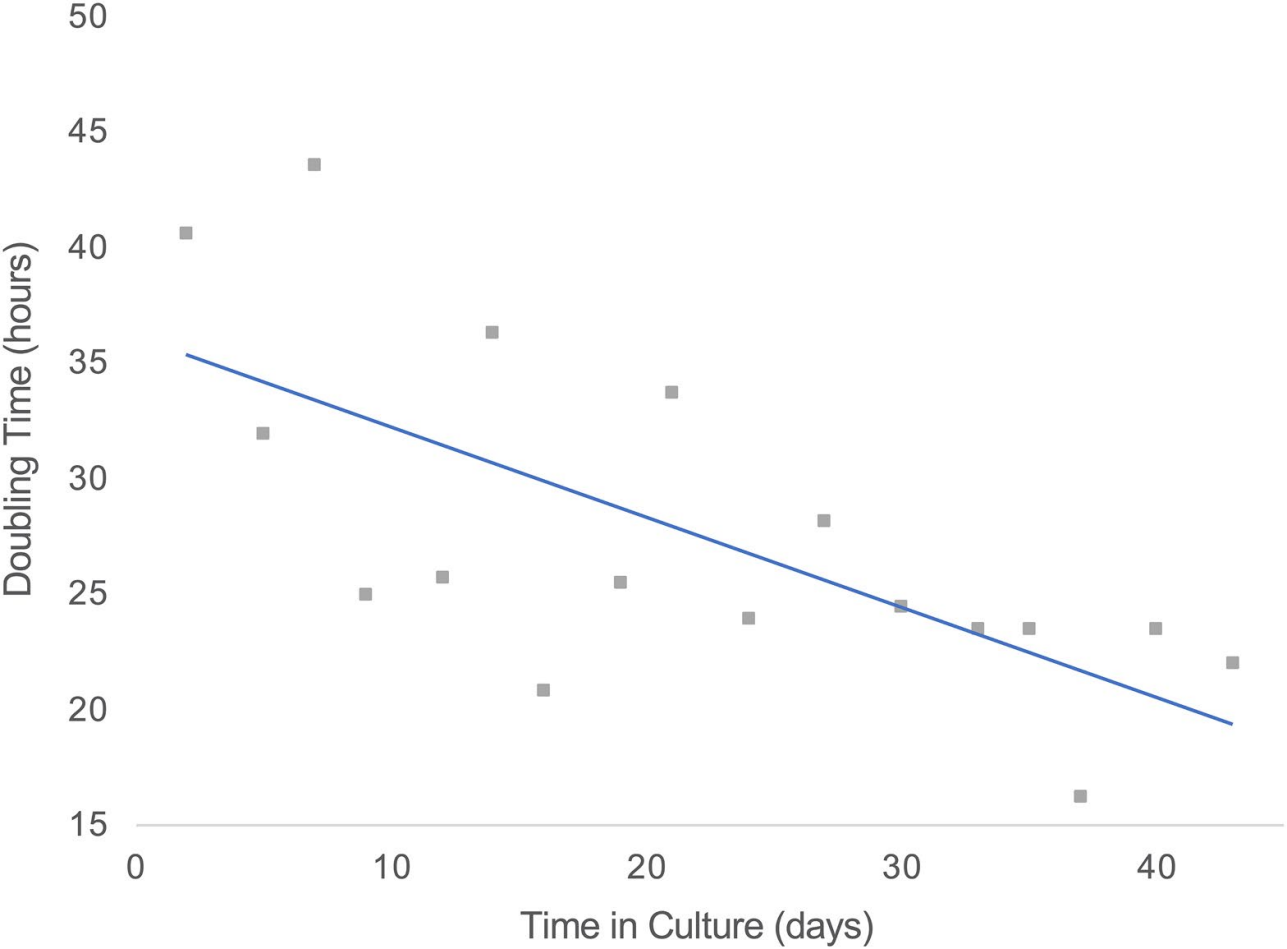
Jurkat proliferation rate—Continuous culture of transduced Jurkat cultures revealed a gradual increase in their proliferation rate (ergo decrease in doubling time). Mean viable cell counts from all 4 replicates at the end of each 2–3 days culture period are shown for each time point.

Jurkat clone JKM1 cells were transduced with the CL3c lentiviral library and cells harvested 24 h later (d0, JCPZ). These cells were used to evaluate the proportion of our CL3c library that could be successfully transduced into mammalian cells. A series of functional selection experiments followed in which JKM1 cells transduced as above were harvested following 47 days of continuous culture (47d, MFZ). Culture of transduced Jurkat cells over a period of 47 days produced a progressive increase in their rate of proliferation (Fig. 3). This increase was confirmed through directly comparing the proliferation of the 4 replicate cell populations, A–D (doubling time 19.1 h (SD (Standard Deviation) 1.6, n = 4), with standard Jurkat JKM1 control populations that had not received library (doubling time 26.3 h (SD 3.7)), (p = 0.01). To examine the potential role of apoptosis resistance as a mechanism underlying selection during prolonged culture, we induced Jurkat cell death through ligation of the Fas cell surface receptor, the classical experimental system for the investigation of caspase-dependent apoptosis in T-cells^58,69–71^*.* 4 × 10^7^ cells from each replicate (d47, MFZ A–D) were treated with a cytotoxic anti-Fas antibody for 96 h (20 ng/mL) (47d + anti-FAS, MF). Wild-type JKM1 cells do not survive such selection with anti-Fas IgM^58^. Anti-Fas resistance appeared stable in cultures selected with anti-Fas antibody. Notably, a secondary challenge with 20 ng/mL anti-Fas antibody resulted in 62.8% (SD 17.6) survival after 22 h for the replicates A–D (d47 + anti-FAS, MF), compared to 2.5% (SD 1.2) for control cells.

Our custom sequencing and bioinformatic approaches (see “Materials and methods” section) were used to characterise the complexity of inserts present and the extent of genome coverage achieved by the CL3c lentiviral library, evaluate our ability to transduce the entire library into Jurkat mammalian cells, and to determine the effects of functional selection on the sequences remaining within our selected cell populations. Sequencing data were generated at all points throughout the study from the initial Genomic Digest, CL3c library and Jurkat cells following transduction (d0, JCPZ), to the highly selected cell populations following continuous culture (d47, MFZ) and continuous culture plus anti-FAS IgM (d47 + anti-FAS, MF).

### Read-level analysis

Our amplicon sequencing approach generated an average of ~ 34 million mapped read pairs per sample (range 24.6–49.7 million read pairs). Our initial approach considered how these mapped reads were distributed across the human genome by separating the human reference genome (hg38) into 1 kb bins (of which there were 3207891) and determining whether each bin included a mapped read or not. This approach was designed to rapidly appraise where in the genome our insert populations were derived from, and whether there was any obvious reduction in diversity following selection. Genomic bin counting demonstrated that > 85% of the human genome was represented in our lentiviral library (CL3c _1 and CL3c_2 merged) and confirmed that we were able to transduce up to 88% of these sequences into the Jurkat JKM1 cell line (JCPZ d0). These data confirm that our CL3c library is both highly diverse, and that we were able to transduce Jurkat JKM1 cell populations with very many sequences simultaneously (Table 1).

**Table 1:** The entire human genome reference (hg38) was split into 3,207,891 1-kilobase genomic bins.

Experimental group	1 kb bins filled (%)	Range (%)
Genome digest	92.91	91.84–93.97
CL3c library	78.81	77.19–80.42
JCPZ (d0)	65.16	57.57–74.79
MFZ (47d continuous culture)	63.65	60.15–65.87
MF (47d continuous culture + anti-FAS IgM)	49.16	31.64–92.3*
MF (excluding MF_D*)	34.78	31.64–40.01
Genome digest merged*	94.63	–
CL3c library merged*	85.26	–

Using multiBamSummary (version 3.3.2), the number of bins that contained ≥ 1 mapped read(s) was determined and expressed as a percentage relative to the total number of genomic bins available.

*Merged datasets were produced by combining bam files from each respective replicate (samtools merge) immediately following mapping.

**Figure 4:**
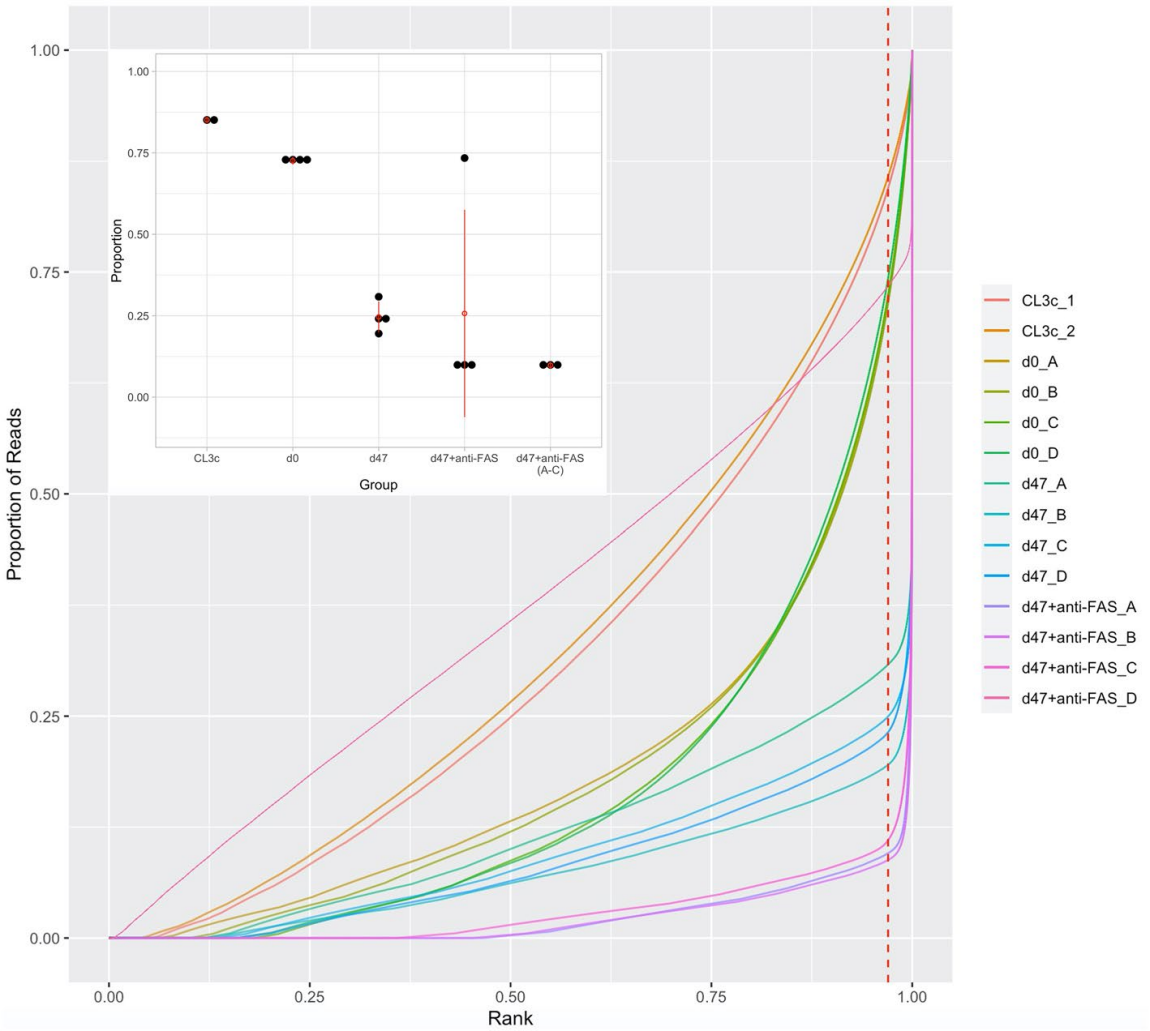
BAMFingerPrint analysis—Proportion of the entire human genome covered by sequence data is shown. Considering reads contained within 97% of all genomic bins (inset panel) revealed that > 85% (84.5–85.8) and 72.6% (71.4–74.3) of the total read count was reached for the CL3c samples (library before transduction) and JCPZ, d0 samples suggesting a broad distribution of reads from across the entire genome. In contrast, just 24.6% of the total read count was reached for those cells subjected to continuous culture (d47, MFZ) consistent with marked selection.

In order to investigate how the sequencing reads mapped across the genome, and in doing so to explore evidence of reduced sequence diversity in our selected samples, a BAMFingerPrint analysis approach was used. In the absence of selection, one expects a broad distribution of reads across the genome, whereas following selection, one expects focused areas of coverage overlaying those areas of the genome with functional capability. Considering reads contained within 97% of all genomic bins (Fig. 4 red dotted line) revealed that > 85% (84.5–85.8) and 72.6% (71.4–74.3) of the total read count was reached for the CL3c samples (library before transduction) and JCPZ, d0 samples suggesting a broad distribution of reads from across the entire genome. In contrast, just 24.6% of the total read count was reached for those cells subjected to continuous culture (d47, MFZ) consistent with marked selection. Strikingly, less than 10% of the total read count was reached when cells were treated with anti-FAS IgM post continuous culture (d47 + anti-FAS, MF_NoD), consistent with even more marked sequence selections. These data support our hypothesis that selection reduces the population of sequences to only those with demonstrable function i.e. the ability to evade otherwise lethal stimuli. It is noteworthy that MF (d47 + anti-Fas) replicate D appeared as an outlier in this analysis, and we make provision for this moving forward by including group level data for d47 + anti-FAS, MF samples both with “MF” and without “MF No_D” this replicate for transparency.

### Insert-level analysis

We next considered our mapped reads as “insert sequences” by identifying regions of increased read coverage on the genome using MACS2 call peak and using these to define the bounds of library inserts. This enabled us to effectively assemble individual reads into “units” with potential function, and was necessary given the sequencing reads generated were shorter than our insert size distribution i.e. they were not full-length. The genomic coordinates corresponding to peaks in sequence coverage across the entire human genome were converted into bed format and the number of reads mapping to these defined locations determined in each sample using bedtools multicov version 2.29.2.

We identified 845,461 different inserts across our experimental sample set, with a size range of between 132 and 8293 bp. In considering reads mapped specifically to inserts, our initial genome digest sample attracted the least reads (7.25 million), as expected, given this comprised input DNA only and was not the result of a targeted PCR amplification step as applied to the remaining samples. Sequencing reads in the selected samples (d47, MFZ and d47 + Fas, MF) were clearly more focused in discrete areas of the genome than in the unselected samples (Fig. 4), even though the total number of reads mapping to inserts was similar (Table 2).

**Table 2:** The number of reads mapping to all inserts for each experimental group.

Experimental group	Mean mapped reads (Millions)	S.E.M
Genome digest	7.25	1.87
CL3c library	17.43	1.82
JCPZ (d0)	17.87	1.27
MFZ (47d continuous culture)	21.20	3.08
MF (47d continuous culture follwed by anti-FAS IgM)	19.86	2.61
MF (excluding MF_D*)	22.02	2.07

We next considered the diversity of inserts present within our CL3c library, starting transduced host cell population (d0, JCPZ), and highly selected cell populations. The expression of each insert was normalised to the total number of mapped reads in each sample to account for variation in sequence coverage, and the expression of each insert expressed in terms of “reads per million reads mapped”. The chromosome start and stop position of each insert was added and the nominal insert length calculated.

An insert was considered to be present in each sample when 10 or more reads mapped to that location. The CL3c library contained in excess of 485,000 different insert sequences with the required sequence coverage. Approximately 338,000 inserts were successfully transduced into our starting cell population (d0, JCPZ), representing ~ 70% of all inserts in the CL3c library. In considering the impact of selection upon insert diversity, continuous culture (d47, MFZ) reduced the number of insert sequences by ~ 77% and continuous culture + anti-FAS IgM treatment (d47 + anti-FAS) by ~ 90%. These data are consistent with marked functional selection (Table 3), revealing only those sequences that enabled cells to survive the selection conditions.

**Table 3:** The number of different inserts identified in each experimental group.

Experimental group	Inserts (mean)	S.E.M	%Reduction
Genome digest	294,907.00	97,030.00	
CLC3 library	485,827.00	26,833.00	
JCPZ (d0)	337,861.50	9140.92	30.46
MFZ (47d continuous culture)	76,900.75	2069.83	77.24
MF (47d continuous culture + anti-FAS IgM)	43,430.75	50,235.00	87.15
MF (excluding MF_D*)	26,838.00	5878.10	92.06

**Figure 5:**
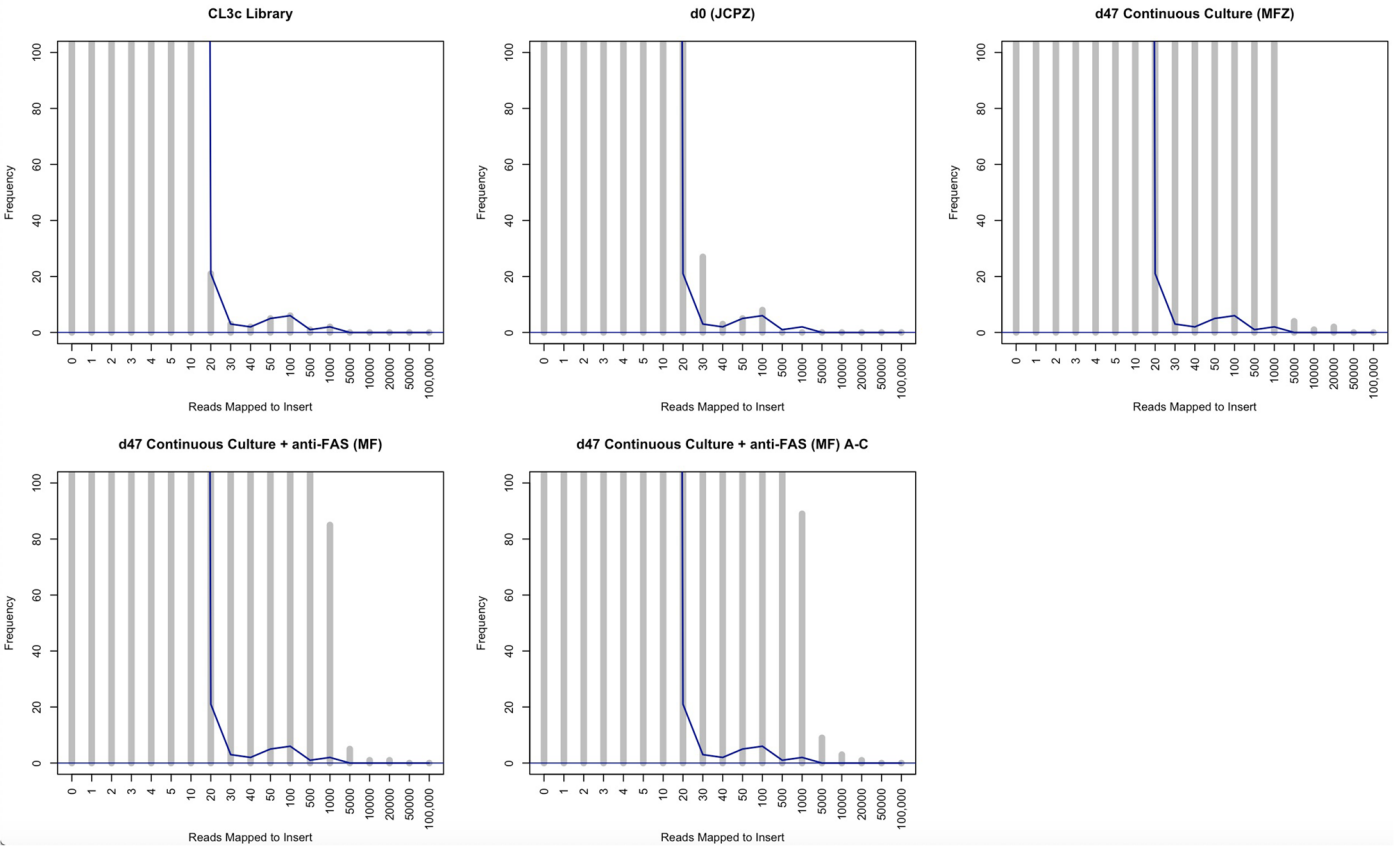
Frequency of inserts at defined coverage levels—Initial library (CL3c) and d0 transduced (JCPZ) samples presented relatively homogenously with the vast majority of inserts having a coverage of 20 or less reads per million. In contrast, the selected cell populations (d47 MFZ and d47 + anti-FAS MF) presented with a large increase in focused presence, and a very large increase in the maximal presence values noted. Maximal presence was increased dramatically in the selected sample sets; CL3c (1466), d0 (433), increasing to d47, MFZ (32,555), d47 + anti-FAS (28,055) [d47 + anti-FAS, MF_NoD (36,988)], suggesting the existence of large cell sub-populations that harbour a specific insert conferring a proliferation/survival advantage. The frequency of inserts at each coverage level in the CL3c library is indicated on all panels by way of a blue line to enable comparison between the initial library and the selected samples. The Y axis terminates at a frequency of ≥ 100 for clarity of data presentation—complete data presented in Supplementary Fig. S5.

We next considered the number of reads mapping to each insert as a proxy of the number of cells harbouring that same insert within each cell population. In this regard, the more reads mapping to a given insert sequence indicated a greater presence of cells in the cell population with that insert, and potentially the importance and/or functional potency of the insert. Mean insert presence remained stable irrespective of experimental condition at approximately 1.18 reads per million mapped reads per insert, for CL3c, d0, d47, and d47 + anti-FAS, respectively. Median values revealed a higher presence per insert in the starting library (CL3c = 0.69) and d0 samples (d0, JCPZ = 0.40) than the selected sample sets d47, MFZ (0.067) and d47 + anti-FAS, MF (0.122) [d47 + anti-FAS, MF_NoD (0.133)]. The most striking results however related to the range of insert presence, in particular maximal presence, in each context. These data revealed specific inserts in the selected populations that were present in very many cells—indicating that they afforded a clear survival advantage. Maximal presence was increased dramatically in the selected sample sets; CL3c (1466), d0 (433), increasing to d47, MFZ (32,555), d47 + anti-FAS (28,055) [d47 + anti-FAS, MF_NoD (36,988)], suggesting the existence of large cell sub-populations that harbour a specific insert conferring a proliferation/survival advantage. As demonstrated below in Fig. 5, insert presence was far less focused in the library (CL3c) and starting cell population (d0, JCPZ) in comparison with the selected cell populations. CL3c and d0 JCPZ samples present relatively homogenously across the range of presence values with no obvious “peaks” in presence. In contrast the selected cell populations (d47 MFZ and d47 + anti-FAS MF) present with a large increase in focused presence, and a very large increase in the maximal presence values noted. Complete frequency data (log Y axis) is shown in Supplementary Fig. S5.

### Annotation and prioritisation of functionally selected insert sequences for further analysis

The data matrix containing insert presence data (normalised as above for sequencing depth), was complemented with a range of additional data to aid interpretation and prioritisation, using a range of custom scripts. The coordinates of restriction enzyme sites (Dra1 and Aan1) were extracted from the human genome and the distance between each insert and a suitable site calculated using the BEDOPS closest-features algorithm. Using the same approach, the distance between each insert sequence and the following features was determined; known features from the GENCODE GRCh38.p13 comprehensive gene annotation superset in GTF format (at the gene, intron and exon level) available here:

https://ftp.ebi.ac.uk/pub/databases/gencode/Gencode_human/release_38/gencode.v38.chr_patch_hapl_scaff.annotation.gtf.gz, entries in the LNCipedia database version 5.2^72^ (standard and high confidence), lncRNAs identified as part of the Cancer lncRNA census dataset^1^, and lncRNAs identified by Liu^73^ and Sarropoulos^50^. In addition, a range of calculated fields were added to assist in prioritisation, including reporting the number of independent replicates each insert was identified in, the presence of “directional reads” containing sequence information derived from the forward PCR primer and thus reads retaining the ability to orientate a given insert, and finally, potentiation scores for 47d and 47d + anti-FAS (calculated as the mean expression of each insert at d47 or 47d + anti-FAS, divided by the mean of each insert at d0, JCPZ expressed as a percentage. A separate calculation excluding replicate MF_D was conducted and labelled 47d + ant-FAS_NoD.). These operations were performed in RStudio.

Of the 845,461 inserts identified across all samples, 741,912 (87.7%) had a Dra1 or Aan1 site located within 100 bp, 517,732 (61.2%) were supported by directional reads, and 514,807 (60.9% of the total library) had both a suitable restriction enzyme site AND directional read support, herein termed “High Confidence Inserts”. Considering the 845,461 inserts identified across all samples, 522,106 mapped within 100 bp of known genes recorded in the GENCODE GRCh38.p13 comprehensive gene annotation superset. The vast majority of inserts were located within the introns of genes (497,159) with a smaller proportion (85,382) located within exons. By a process of elimination, we identified those inserts that were intergenic on the basis they did not appear within the GENE level annotation file (which naturally encompasses both EXON and INTRON data); intergenic reads accounted for ~ 38% of our total library. Considering reads that were located within 1000 bp of known genes, and therefore within potential gene promoter regions, identified an addition 12,615 inserts (1.5%).

Biotype analysis revealed 361,469 library sequences mapped to protein coding genes, and 142,525 to lncRNAs. Constraining our analysis to exonic regions only revealed 57,459 insert sequences mapped to protein coding genes and 18,960 to lncRNAs, respectively. Comparable results were obtained for both the entire and high confidence datasets. Results of the annotation routine are shown in Table 4.

**Table 4:** Annotation of all library inserts (unselected).

Feature	All inserts (845,461)	High confidence inserts (514,807)
Gencode genes	522,106 (62%)	328,386 (64%)
Gencode exons	85,382 (10%)	55,777 (11%)
Gencode introns	497,159 (59%)	313,598 (61%)
Intergenic	323,355 (38%)	186,421 (36%)
LNCipedia (V5.2)^72^	257,996 (30%)	160,962 (31%)
LNCipedia (V5.2_HC)^72^	228,570 (27%)	143,279 (28%)
Cancer lncRNA Census^1^	9175 (1.1%)	5706 (1.1%)
Liu et al.^73^	3029 (0.36%)	1864 (0.36%)
Sarropoulos et al.^50^	34,431 (4%)	22,767 (4.4%)

To identify functional sequences selected in response to continuous cell culture alone (d47, MFZ) and continuous cell culture followed by anti-FAS treatment (d47 + anti-FAS, MF), we prioritised inserts selected in at least 3 independent experimental replicates, and with evidence of directional read support (confirming that the insert sequences were indeed library-derived on the basis that they contained vector sequence prior to trimming). This procedure reduced the many thousands of insert sequences to; 3895, 3778 and 3808 selected inserts for the d47, d47 + anti-FAS, and d47 + anti-FAS_NoD conditions respectively. Biotype analysis was conducted to determine the classification of those sequences that mapped to known genes following comparison with the GENCODE GRCh38.p13 comprehensive gene annotation superset. Of the 3895 d47, MFZ selected sequences, 2458 mapped to known genes; of these, 1653 were protein coding genes and 716 lncRNAs. An additional 64 sequences (2522 in total) were found to be located within 1000 bp of the start of known genes.

Of the 3778 d47 + anti-FAS, MF selected sequences, 2405 mapped to known genes; of these, 1647 were protein coding genes and 678 lncRNAs. An additional 55 sequences (2460 in total) were found to be located within 1000 bp of the start of known genes. In considering only those reads that mapped to exons; 401 mapped to protein coding genes and 181 to lncRNAs in the d47 MFZ condition, and 417 and 174 mapped in the d47 + anti FAS MF condition, respectively. Overall, there was an apparent increase in the number of inserts that mapped to the exons of known gene in the selected cell populations (17%) versus the entire unselected dataset (10%). Annotation information is shown in Table 5 and the resulting datasets are available as Supplementary Tables S1–S3.

**Table 5:** Annotation of the selected insert sequences.

Feature (within 100 bp)	MFZ_Selected (3895)	MF_Selected (3778)	MF_NoD_Selected (3808)
Gencode genes	2458 (63%)	2405 (64%)	2426 (64%)
Gencode exons	643 (17%)	651 (17%)	662 (17%)
Gencode introns	2349 (60%)	2297 (61%)	2315 (61%)
Intergenic	1437 (37%)	1373 (36%)	1382 (36%)
LNCipedia (V5.2)^72^	1296	1264	1271
LNCipedia (V5.2_HC)^72^	1167	1127	1133
Cancer lncRNA Census^1^	43	40	40
Liu et al.^73^	26	25	25
Sarropoulos et al.^50^	320	308	313
Undescribed*	1110 (28.5%)	1068 (28.3%)	1073 (28.1%)

*Potential novel (undescribed) inserts were identified on the basis that they lacked any annotation in the databases considered herein.

The d47, MFZ selected dataset (cells subjected to 47d continuous culture) included 43 lncRNAs with known causal roles in cancer including *MIR100HG*, *AZIN1*-*AS1*, *HULC*, *SAMMSON*, *HOXB-AS3*, and *SOX9-AS1*. Similarly, the d47 + anti-FAS, MF dataset included sequences derived from 40 lncRNAs with known causal roles in cancer including *SChLAP1*, *DDN-AS1*, *PCAT-1*, *SOX2OT*, *DLEU1*, and *PCA3* (the 6 most abundant in each case)—on occasion, different fragments of the same lncRNA were identified, potentially demonstrating the parts thereof required for function. The presence of established cancer associated lncRNAs within our dataset, identified by a range of unrelated techniques, adds significant support to our more novel approach. After all, one expects our approach to identify known as well as novel sequences.

Intersecting the three datasets (Table 5) allowed the identification of sequences present in both the MF and MFZ (536), MF_NoD and MFZ (538), and MF and MF_NoD (3772) conditions. The MF and MF_NoD datasets were very similar (99% similar) in terms of the sequences prioritised, despite replicate D appearing as an outlier during the bin-level analysis (Fig. 3). Furthermore, approximately 13% of sequences originally selected in the d47, MFZ condition were subsequently re-selected following the induction of apoptosis by anti-Fas antibody (d47 + anti-FAS, MF), suggesting that apoptosis resistance may indeed act as the mechanism for selection by continuous culture, for this significant minority of inserts. Annotation information is shown in Table 6 which describes whether the sequences identified in our experiments have been described by others. It is noteworthy that these comparisons are based upon inserts identified on the basis that they were present in at least three independent replicates and supported by evidence of directional read support. When one considers inserts with *any* evidence of inclusion (i.e. the presence of reads mapping to those loci), then in excess of 99% of inserts present in the MF condition were present in the MFZ condition, as expected.

**Table 6:** Annotation of the selected insert sequences common across the experimental conditions presented below.

Feature (within 100 bp)	MF and MF_NoD common (3772)	MF_ and MFZ common (536)	MF_NoD and MFZ common (538)
Known hg38	2403	330	334
LNCipedia (V5.2)^72^	1262	161	162
LNCipedia (V5.2_HC)^72^	1125	146	147
Cancer lncRNA Census^1^	40	4 (11^1^)	4 (11^1^)
Liu et al.^73^	25	4	4
Sarropoulos et al.^50^	308	42	43
Undescribed*	1075	165	163

^1^When considering inserts contained within the same lncRNA, but not necessarily in the exact same genomic location.

*Potential novel (undescribed) inserts were identified on the basis that they lacked any annotation in the databases considered herein.

Four sequences derived from lncRNAs with known causal roles in cancer were selected in both the d47, MFZ and d47 + anti-FAS, MF conditions (AC084816.1*^1^, AC087473.1^1^, MIRLET7BHG*^1^, and TP53TG1^42–45^) and as we have observed previously, multiple fragments of certain lncRNAs were independently selected, potentially highlighting the functional elements of these lncRNAs. Considering selection at the gene level, rather than based upon the isolation of identical sequences contained within, we identified 11 lncRNAs with known causal roles in cancer selected in both the d47, MFZ and d47 + anti-FAS, MF conditions including AC084816.1*^1^, *AC097103.2*^2^, *AC087473.1*^1^, CASC15*^3–7^, DLEU1*^8–13^, *ENTPD1*-AS1*^1^, HULC*^14–22^, MIRLET7BHG*^1^, *PCAT-1*^23–33^, *SChLAP1*^34–41^, and *TP53TG1*^42–45^. The presence of these sequences in the anti-Fas selected populations (d47 + anti-Fas, MF) suggests that suppression of apoptosis is one mechanism that contributes, not only to their selection by continuous culture, but also, potentially, to their association with cancer. Several of these genes have indeed been implicated in apoptosis control by independent laboratories^39,45,74^. It is striking that multiple functional sequences were identified from within many of these lncRNAs, for example; three functional sequences derived from PCAT-1 were independently selected (2 × selected in 3 MFZ independent replicates and 1 selected in the MF condition). On the other hand, the same specific functional sequence was identified at least 6 times for TP53TG1 (3 × MFZ and 3 × MF). These data emphasise the power of this strategy to identify functional genes and may also help to reveal the specific elements/minimal composition of each lncRNA required for function. Cancer lncRNAs previously implicated in cancers of the blood are denoted with an asterisk.

In order to gain insight into the performance of a given insert in each experimental condition, we calculated the MF to MFZ ratio, indicative of the proportion of cells harbouring each insert following anti-FAS selection (d47 + anti-FAS, MF), relative to their presence in the continuous culture condition (d47, MFZ). Inserts present in the MFZ condition were, on average, present in the MF condition at ratio of 2.66:1 i.e. the inserts were 2.66 times more abundant on re-selection. Considering only those inserts that met the criteria for inclusion in our selected insert datasets (present in at least three independent replicates and with evidence of directional read support) for both the d47, MFZ and d47 + anti-FAS, MF conditions revealed a ratio of > 10.5:1, suggesting that these inserts were more strongly selected.

### Independent validation of novel cell fate modifying sequences

Five selected sequences (Table 7) were independently validated on the basis of their selection in both the MFZ and MF conditions, by producing plasmids (pcDNA3.1sense backbone) containing each chemically synthesised insert of interest, and used to transduce naive Jurkat JKM1 cells, followed by challenge with anti-Fas IgM (see “Materials and methods” section). Viable cell numbers relative to identically treated control cells receiving empty plasmid were determined. Sequence composition of the validation plasmids is included within Supplementary Table S4.

**Table 7:** Sequences subjected to independent validation using custom pCDNA3.1 plasmids.

Validated insert location	MFZ potentiation (%)	MF potentiation (%)	Details
Chr13: 24,089,729–24,090,017	108.3	4619.7	Within SPATA13
Chr7: 137,120,513–137,121,580	695.2	7344.3	lnc-PTN-2:10^72^
Chr5: 102,160,152–102,160,734	17,877.4	9400.3	Not Described
Chr5: 154,258,210–154,258,883	12,371.4	6124.2	Intron GALNT10
Chr5: 137,315,818–137,316,592	316.7	242.1	lnc-SPOCK1-1:2^72^

**Figure 6:**
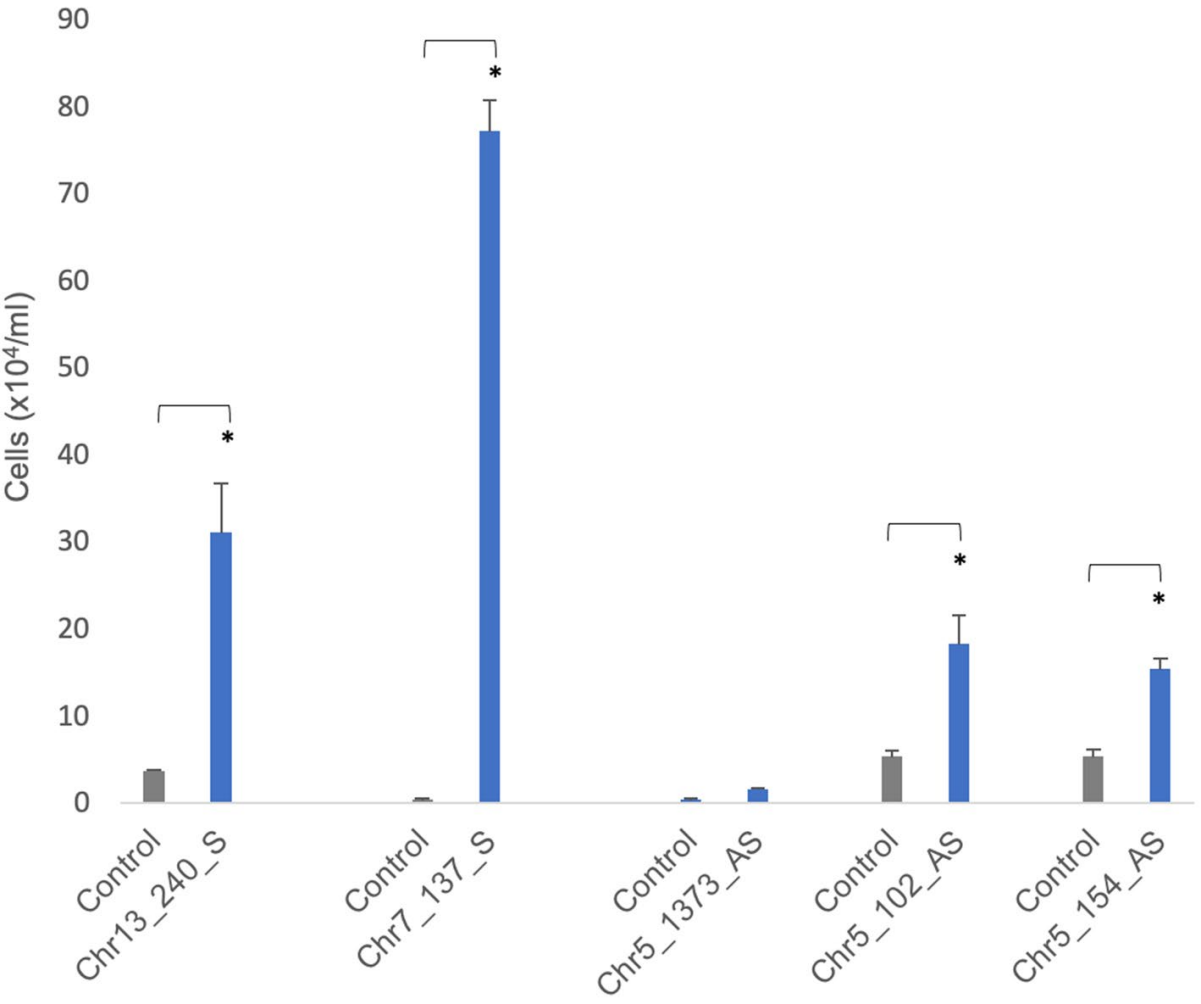
Independent validation—Jurkat cells (5 × 10^5^ cells/ml) were transfected with our candidate functional sequences (see Table 7) in custom pcDNA3.1 plasmids and challenged with 10–20 ng/ml anti-Fas IgM. Viable (Trypan blue-excluding) viable cell concentrations were determined 7 to 18 days after the addition of antibody (see “Materials and methods” section). The mean viable cells ± standard deviation of 3 replicate cultures are shown. In 4 out of 5 cases, transfected cultures were significantly increased over empty vector transfected controls run in parallel (*p < 0.05).

Four out of the five hits subjected to independent validation conferred significant resistance to anti-FAS IgM mediated apoptosis, evidencing that the sequence alone was sufficient for function (Fig. 6). Whilst the mean number of cells in the Chr5:1373 transfected and challenged populations was numerically larger than controls, this observation was not statistically significant nor was the effect size anywhere near that of the other four validated hits.

## Concluding remarks

Investigation of long non-protein coding RNAs (lncRNAs) has become a key area in biological and biomedical research. Although the analysis of this vast number of transcripts is still at an early stage, it is evident that many lncRNAs play crucial roles in molecular cell biology. Demonstrations of the functional importance of individual RNAs are impressive and growing, however the vast majority—many thousands—of lncRNAs are still entirely uncharacterised, and some of these currently uncharacterised ncRNAs are likely to play critical roles in the control of cell behaviour that have yet to be revealed. There is thus a need for high-throughput approaches that enable the identification of key lncRNAs from across entire genomes (unrestricted by the physiological expression profiles of specific lnRNAs in specific tissues/biological contexts), based upon demonstrable function. This latter requirement ensures that lncRNAs identified through such studies are actively responsible for the effect seen, for example the evasion of apoptosis, as opposed to being passively modulated as a consequence of changes caused by other genes.

Herein we report a genome-scale screening approach that overcomes the limitations imposed by the highly tissue-specific expression of lncRNAs by interrogating sequences from the whole genome. Combining our established forward genetics approach with next generation sequencing and bioinformatics, we identify many thousands of lncRNAs from across the breadth of the genome, based entirely upon their function. The power of this strategy was confirmed both by multiple hits on established cancer genes, and by confirmation of the resistance conferred by selected identified sequences (Fig. 6), suggesting that, although this screen, like all other screens, produces some false positives, these are not highly represented among the strongest hits.

These data alone represent a significant contribution to the identification of lncRNAs involved in the regulation of apoptosis. Our strategy represents a marked step-change in the rate of functional lncRNA discovery and can be applied to an almost endless range of situations through modification of the selection regime and/or cell types used. Whilst our current investigation utilises a human genomic DNA library applied to human cells, there is further scope to apply this strategy to the functional discovery of other lncRNAs in other species e.g. key model species used in drug discovery, and also in the investigation of conservation of function during evolution through the application of a library to cells from a different species. The identification of lncRNAs that control cell fate is essential to our understanding of molecular cell biology as a whole, and many opportunities in diverse fields are likely to result from our approach and initial data.

## Supplementary Information

Supplementary Legends.

Supplementary Table S1.

Supplementary Table S2.

Supplementary Table S3.

Supplementary Table S4.

Supplementary Figure S5.

## Data Availability

The datasets generated during and/or analysed during the current study are available from the corresponding author on reasonable request. Data are also stored in the NIH Sequence Read Archive under project submission SUB10570351. An annotated script has been deposited on the GitHub website which can be accessed here: https://github.com/dptonge/CL3c_Paper_1/blob/main/CL3c_Paper_1_Script.
