# Supplementary material for: Whole-genome-scale identification of novel non-protein-coding RNAs controlling cell proliferation and survival through a functional forward genetics strategy
Source: Sci Rep. 2022 Jan 7;12:182. doi: 10.1038/s41598-021-03983-5 (PMC8741825; doi:10.1038/s41598-021-03983-5)
Supplement: Supplementary file 1 — Supplementary Legends. [file 41598_2021_3983_MOESM1_ESM.docx]

**Supplementary Data**

**Supplementary Table S1**

Sequences selected following 47d of continuous culture (d47, MFZ); inserts were triaged on the basis of being selected in at least 3 independent experimental replicates (MFZ A – D), and with evidence of directional read support (confirming that the insert sequences were indeed library-derived on the basis that they contained vector sequence prior to trimming). Columns D – F report the number of experimental replicates in which each insert was selected. Potentiation scores (Columns N-P) for 47d and 47d + anti-FAS were calculated as the mean expression of each insert at d47 or 47d + anti-FAS, divided by the mean of each insert at d0, JCPZ expressed as a percentage. Column AT reports the mean number of directional reads per million (defined above) supporting each insert.

**Supplementary Table S2**

Sequences selected following 47d of continuous culture + anti-FAS treatment (d47+antiFAS, MF); inserts were triaged on the basis of being selected in at least 3 independent experimental replicates (MF A – D), and with evidence of directional read support (confirming that the insert sequences were indeed library-derived on the basis that they contained vector sequence prior to trimming). Columns D – F report the number of experimental replicates in which each insert was selected. Potentiation scores (Columns N-P) for 47d and 47d + anti-FAS were calculated as the mean expression of each insert at d47 or 47d + anti-FAS, divided by the mean of each insert at d0, JCPZ expressed as a percentage. Column AT reports the mean number of directional reads per million (defined above) supporting each insert.

**Supplementary Table S3**

Sequences selected following 47d of continuous culture + anti-FAS treatment – replicates A – C only (d47+antiFAS, MF_NoD); inserts were triaged on the basis of being selected in at least 3 independent experimental replicates (MF_NoD A – C), and with evidence of directional read support (confirming that the insert sequences were indeed library-derived on the basis that they contained vector sequence prior to trimming). Columns D – F report the number of experimental replicates in which each insert was selected. Potentiation scores (Columns N-P) for 47d and 47d + anti-FAS were calculated as the mean expression of each insert at d47 or 47d + anti-FAS, divided by the mean of each insert at d0, JCPZ expressed as a percentage. Column AT reports the mean number of directional reads per million (defined above) supporting each insert.

**Supplementary Table S4**

Nucleic acid sequences for the validation plasmids used in the generation of Fig.6. Sequences were chemically synthesised and inserted into pcDNA3.1 sense expression plasmids (GenScript Limited).

**Supplementary Figure S5**

Frequency of inserts at defined coverage levels – Initial library (CL3c) and d0 transduced (JCPZ) samples presented relatively homogenously with the vast majority of inserts having a coverage of 20 or less reads per million. In contrast, the selected cell populations (d47 MFZ and d47+anti-FAS MF) presented with a large increase in focused presence, and a very large increase in the maximal presence values noted. Maximal presence was increased dramatically in the selected sample sets; CL3c (1466), d0 (433), increasing to d47, MFZ (32,555), d47+anti-FAS (28,055) [d47+anti-FAS, MF_NoD (36,988)], suggesting the existence of large cell sub-populations that harbour a specific insert conferring a proliferation/survival advantage. Note: the frequency of inserts at each coverage level in the CL3c library is indicated on all panels by way of a blue line to enable comparison between the initial library and the selected samples. Note: Logarithmic Y axis.
