# Supplementary material for: Whole-genome-scale identification of novel non-protein-coding RNAs controlling cell proliferation and survival through a functional forward genetics strategy
Source: Sci Rep. 2022 Jan 7;12:182. doi: 10.1038/s41598-021-03983-5 (PMC8741825; doi:10.1038/s41598-021-03983-5)

Supplementary Figure S5

Frequency of inserts at defined coverage levels – Initial library (CL3c) and d0 transduced (JCPZ) samples presented relatively homogenously with the vast majority of inserts having a coverage of 20 or less reads per million. In contrast, the selected cell populations (d47 MFZ and d47+anti-FAS MF) presented with a large increase in focused presence, and a very large increase in the maximal presence values noted. Maximal presence was increased dramatically in the selected sample sets; CL3c (1466), d0 (433), increasing to d47, MFZ (32,555), d47+anti-FAS (28,055) [d47+anti-FAS, MF_NoD (36,988)], suggesting the existence of large cell sub-populations that harbour a specific insert conferring a proliferation/survival advantage. Note: the frequency of inserts at each coverage level in the CL3c library is indicated on all panels by way of a blue line to enable comparison between the initial library and the selected samples. Note: Logarithmic Y axis.

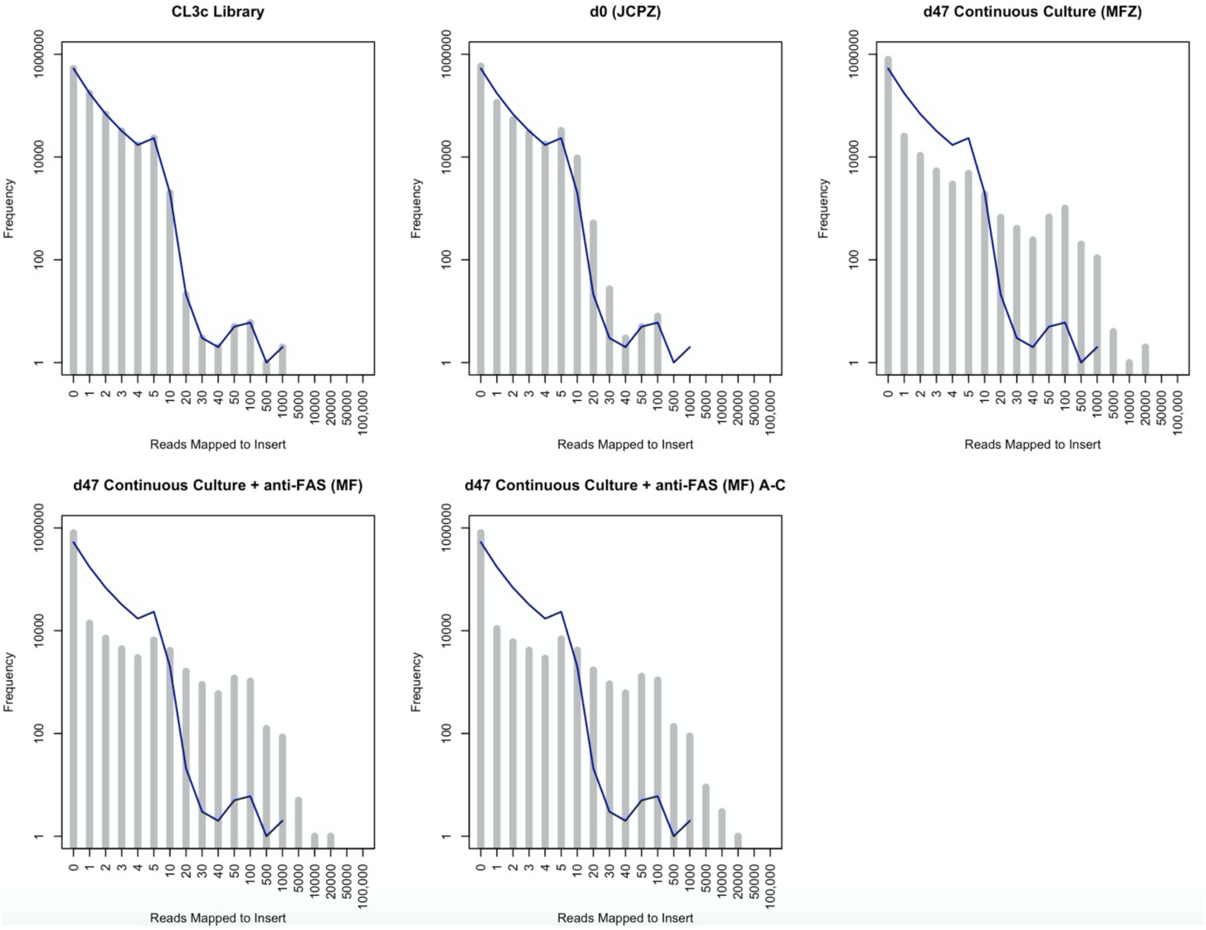

Supplement: Supplementary file 6 — Supplementary Figure S5. [file 41598_2021_3983_MOESM6_ESM.pdf]
